# Estrogen Exposure is Associated With Reduced Otosclerosis Risk in Obesity and Hormone Therapy

**DOI:** 10.1097/ONO.0000000000000083

**Published:** 2026-01-22

**Authors:** Maia Smith, Sudhanvan Iyer, Delaney E.S. Clark, Simon Warren, Brian McKinnon

**Affiliations:** 1 John Sealy School of Medicine, The University of Texas Medical Branch, Galveston, TX; 2 Department of Otolaryngology, The University of Texas Medical Branch, Galveston, TX.

**Keywords:** Estrogen, Hearing Loss, Metabolism, Middle Ear, Otosclerosis

## Abstract

**Objective:**

To examine whether conditions associated with elevated-estrogenic states, including obesity, type 2 diabetes mellitus (T2DM), and postmenopausal estradiol hormone replacement therapy (HRT), are associated with the development of otosclerosis.

**Methods:**

We performed 3 retrospective cohort analyses using the US TriNetX network. Population included adults ≥18 years of age with an ear evaluation (the International Classification of Diseases [ICD]-10 Z01.1). Exposures included obesity (ICD-10 E66), T2DM (ICD-10 E11), and HRT (RxNorm 4083). Primary outcome of interest was otosclerosis (ICD-10 H80). Exclusion criteria for obesity and T2DM cohorts included type 1 diabetes (ICD-10 E10); for all cohorts, criteria included congenital ear abnormalities (ICD-10 Q16) or outcome of interest ≥20 years ago. 1:1 propensity score matching was performed on each cohort for demographics, metabolic comorbidities, ototoxic medications, lifestyle, body mass index, age, and sex. Relative risk with 95% confidence intervals was calculated; *P* < 0.05 represents statistical significance.

**Results:**

Otosclerosis occurred in 25 individuals with obesity with 52 controls (risk ratio [RR] = 0.48, *P* < 0.005). In the T2DM cohort, 15 cases were present, whereas the control population had 27 cases (RR = 0.56, *P* = 0.06). Among the estradiol HRT cohort, 303 cases were present compared with 369 in control (RR = 0.82, *P* < 0.05).

**Conclusions:**

Obesity and estradiol HRT are associated with a decreased risk of developing otosclerosis. A similar but nonsignificant trend was found in patients with T2DM. This suggests a protective role of estrogen against otosclerosis. Future studies may serve to elucidate longitudinal hormone profiling and identify a therapeutic local intervention for individuals with otosclerosis.

Otosclerosis is marked by abnormal remodeling of bone in the otic capsule, resulting in impaired normal transmission of sound, often due to immobilization of the stapes footplate. According to the International Classification of Diseases (ICD-10 H80), otosclerosis involves the formation of spongy bone within the capsule that may progress towards the stapes, leading to fixation, or anteriorly towards the cochlea, resulting in conductive, sensorineural, or mixed hearing loss. It is considered a pathologic condition of the bony labyrinth in which bony ankylosis of the stapes may occur (1). As a result, the stapes is unable to vibrate adequately, leading to progressive conductive and/or sensorineural hearing loss, typically experienced bilaterally. Abnormal bone growth can also occur around the oval window or cochlea, leading to bone resorption and new bone formation, impacting hearing (2). In addition, otosclerosis has been shown to disproportionately affect women at nearly twice the rate of men, with earlier onset and more severe symptoms overall. However, women experience hearing loss later in life than men (beginning at 20–30 years of age in men and 50 years of age in women). Furthermore, literature has demonstrated premenopausal high-frequency hearing in women to be better than men of comparable ages, but postmenopausal high-frequency hearing worse than men of comparable ages (3).

Estrogen is a hormone of great interest in the field of osteobiology due to its vital role in maintaining bone strength and regulation of metabolism, especially in women. It also exhibits significant influence on bone structure, formation, and resorption, as estrogenic effects have demonstrated a protective role against bone resorption by decreasing osteoclast activity in response to Receptor activator of nuclear factor kappa-B ligand and increasing apoptosis of osteoclasts. Clinically, periods of fluctuations in estrogen levels, such as during pregnancy and menopause, have been significantly linked to osteoporosis. Some chronic high-estrogenic states that may influence the careful homeostasis of bone regulation and metabolism include, but are not limited to, obesity, diabetes, and metabolic syndrome (4).

The specific relationship between otosclerosis and estrogen, however, has remained highly inconsistent and controversial in the literature. While some have demonstrated that progestin and estrogen oral contraceptive therapy can damage hearing, other studies have implicated estrogen therapy as a way to potentially slow hearing loss in postmenopausal women (5). In addition, lower levels of G-protein-coupled estrogen receptor-1 (GPER-1) have been found in patients with otosclerosis (6). Based on this strong rationale, we propose a putative protective role of estrogen against the etiopathogenesis of otosclerosis. We hypothesize that elevated-estrogenic states, including obesity, type 2 diabetes mellitus (T2DM), and estradiol hormone replacement therapy (HRT) for menopause, are associated with a reduced risk for otosclerosis.

## MATERIALS AND METHODS

### Study Design and Population

This retrospective cohort study was conducted using the TriNetX Research Network, a global federated health research network that allows access to aggregated, de-identified electronic medical records from approximately 150 million patients across more than 100 health care organizations (HCOs) in the United States. Data available on the TriNetX platform includes de-identified patient materials pertaining to demographics, diagnoses (ICD-10), procedures (common procedural terminology), medications (RxNorm), and laboratory results across a wide variety of clinical settings. The data query was performed through the TriNetX online user interface throughout the months of April to June 2025. No temporal constraints were applied for patient selection.

Three separate analyses were constructed to evaluate the potential associations between (1) obesity (ICD-10 E66), (2) T2DM (ICD-10 E11), and (3) estradiol therapy (ICD-10 N95) for postmenopausal women and otosclerosis. Each analysis featured patients with the diagnosis of interest compared with undiagnosed controls. Minimum patient requirements for selection included age ≥18 years, in addition to at least one encounter documented for ear/hearing evaluation (ICD-10 Z01.1).

### Exposures and Outcomes of Interest

Exposure 1. Obesity and otosclerosis: the experimental group consisted of patients with a diagnosis of overweight or obesity. Obesity was defined using ICD-10 code E66, which includes obesity due to excess caloric intake, drug-induced obesity, and morbid obesity. This code generally corresponds to a body mass index (BMI) ≥30 kg/m^2^ in clinical practice (7). The control group consisted of patients with no ICD-10 code record of overweight/obesity. Both cohorts were refined to exclude patients with congenital ear malformations (ICD-10 Q16) and type 1 diabetes (ICD-10 E10).

Exposure 2. T2DM and otosclerosis: the experimental group consisted of patients with a diagnosis of T2DM. T2DM was defined using ICD-10 code E11, which captures patients with hyperglycemia secondary to insulin resistance (8). The control group consisted of patients with no ICD-10 code record of T2DM. Both cohorts were refined to exclude patients with congenital ear malformations (ICD-10 Q16) and type 1 diabetes (ICD-10 E10).

Exposure 3. Estradiol HRT and otosclerosis: both patient populations of interest consisted of female patients ≥45 years of age with a diagnosis of menopausal or perimenopausal disorders, which includes symptomatic menopause. The estradiol therapy group included women prescribed estradiol (RxNorm 4083). The control group consisted of patients with the N95 ICD-10 code and no RxNorm code for estradiol therapy. Both cohorts were refined to exclude patients with congenital ear malformations (ICD-10 Q16).

The primary outcome of interest for all 3 exposure groups was otosclerosis (ICD-10 H80), which encompasses both otosclerosis and otospongiosis, reflecting the spectrum of disease from early spongiotic bone formation to mature sclerotic foci. This code was selected given its established use for both histopathologic and clinical diagnoses of otosclerosis within the ICD-10 framework (1). Patients with a documented diagnosis of otosclerosis before the index exposure were excluded from all cohort analyses. No temporal restrictions were applied beyond this requirement.

### Baseline Characteristics, Propensity Score Matching, and Statistical Analysis

A 1:1 propensity matching was performed to account for variables including age, sex, race, ethnicity, metabolic comorbidities, ototoxic medications, lifestyle factors, and BMI percentile as indicated in Tables 1–3. Standard mean difference was utilized to assess the balance between matched cohorts and ensure satisfactory matching. The forest plot was generated using Microsoft Excel software.

**TABLE 1.** Comparative analysis for obesity vs no obesity

Demographics		Before propensity score matching						After propensity score matching					
		Obesity (*n* = 99,455)		No obesity (*n* = 619,171)				Obesity (*n* = 34,843)		No obesity (*n* = 34,843)			
		mean ± SD	% of cohort	mean ± SD	% of cohort	Standard difference	*P* value	mean ± SD	% of cohort	mean ± SD	% of cohort	Standard difference	*P* value
AI	Age at Index	44.1 ± 20.1	100.0	42.8 ± 19.6	100.0	0.064	0.000	43.1 ± 20.2	100.0	45.1 ± 20.8	100.0	0.093	0.000
2186-5	Not Hispanic or Latino		71.5		63.9	0.163	0.000		70.7		70.9	0.005	0.505
2106-3	White		63.6		63.7	0.003	0.674		64.5		65.7	0.025	0.001
F	Female		63.4		53.6	0.199	0.000		61.6		61.4	0.004	0.570
M	Male		35.0		43.5	0.176	0.000		36.5		36.7	0.003	0.741
2054-5	Black or African American		21.7		11.8	0.267	0.000		19.7		18.7	0.025	0.001
2135-2	Hispanic or Latino		11.8		8.9	0.095	0.000		11.3		11.8	0.015	0.042
2131-1	Other race		4.3		5.2	0.041	0.000		4.5		4.5	0.002	0.742
2028-9	Asian		2.1		4.4	0.134	0.000		2.4		2.5	0.007	0.338
2076-8	Native Hawaiian or Other Pacific Islander		0.7		0.5	0.027	0.000		0.7		0.7	0.008	0.293
1002-5	American Indian or Alaska Native		0.7		0.7	0.000	0.943		0.6		0.7	0.003	0.740
Diagnoses													
E78	Disorders of lipoprotein metabolism and other lipidemias		44.7		19.8	0.552	0.000		37.3		39.5	0.046	0.000
I10	Essential (primary) hypertension		42.1		17.2	0.566	0.000		34.6		36.7	0.042	0.000
K70-K77	Diseases of liver		11.7		3.5	0.316	0.000		7.5		7.6	0.001	0.931
Z72.0	Tobacco use		5.5		2.5	0.153	0.000		4.4		4.7	0.015	0.055
J44	Other chronic obstructive pulmonary disease		5.3		2.2	0.163	0.000		4.1		4.2	0.006	0.404
F10	Alcohol related disorders		3.7		2.2	0.091	0.000		3.3		3.5	0.013	0.085
Medications													
2555	Cisplatin		0.4		0.3	0.015	0.012		0.3		0.4	0.004	0.566
40048	Carboplatin		0.2		0.2	0.016	0.005		0.2		0.2	0.008	0.290

Eighty-two patients in the obesity cohort and 787 patients in the control cohort were excluded because they met the index event more than 20 years ago. Continuous variables are reported as mean ± SD, and categorical variables as n (%).

n indicates number; N/A, not applicable; SD, standard deviation.

*P* < 0.05 indicates statistical significance.

**TABLE 2.** Comparative analysis for T2DM vs No T2DM

Demographics		Before propensity score matching						After propensity score matching					
		T2DM (*n* = 16,569)		No T2DM (*n* = 106,098)				T2DM (*n* = 15,702)		No T2DM (*n* = 15,702)			
		mean ± SD	% of cohort	mean ± SD	% of cohort	Standard difference	*P* value	mean ± SD	% of cohort	mean ± SD	% of cohort	Standard difference	*P* value
AI	Age at Index	59.2 ± 16.3	100.0	41.6 ± 19.0	100.0	0.991	0.000	58.6 ± 16.4	100.0	59.0 ± 16.5	100.0	0.024	0.036
2186-5	Not Hispanic or Latino		72.4		65.4	0.151	0.000		72.9		73.4	0.012	0.303
2106-3	White		64.1		64.2	0.002	0.784		66.3		67.6	0.027	0.016
F	Female		56.3		56.3	0.001	0.914		55.9		56.1	0.004	0.708
M	Male		41.9		41.0	0.017	0.046		42.2		41.9	0.006	0.575
2054-5	Black or African American		19.5		13.4	0.167	0.000		17.4		17.6	0.006	0.614
2135-2	Hispanic or Latino		12.4		9.3	0.101	0.000		11.3		10.5	0.027	0.017
2028-9	Asian		4.2		3.7	0.028	0.000		4.2		3.9	0.016	0.151
2131-1	Other race		3.9		5.1	0.057	0.000		3.8		3.4	0.021	0.068
2076-8	Native Hawaiian or Other Pacific Islander		1.0		0.5	0.064	0.000		1.0		0.8	0.020	0.076
1002-5	American Indian or Alaska Native		1.0		0.6	0.040	0.000		0.9		0.8	0.011	0.319
Diagnoses													
I10	Essential (primary) hypertension		69.9		18.3	1.217	0.000		68.2		68.3	0.001	0.904
E78	Disorders of lipoprotein metabolism and other lipidemias		68.9		21.2	1.095	0.000		67.2		67.2	0.001	0.933
K70-K77	Diseases of liver		17.6		4.2	0.440	0.000		17.6		10.4	0.208	0.000
J44	Other chronic obstructive pulmonary disease		10.7		2.1	0.357	0.000		10.3		6.8	0.127	0.000
Z72.0	Tobacco use		7.5		2.8	0.214	0.000		6.9		6.8	0.006	0.623
F10	Alcohol related disorders		4.4		2.4	0.111	0.000		4.3		4.5	0.008	0.490
Medications													
2555	Cisplatin		0.4		0.3	0.021	0.008		0.4		0.4	0.003	0.786
40048	Carboplatin		0.4		0.2	0.039	0.000		0.4		0.4	0.004	0.707
Labs													
9084	BMI percentile		1.3		7.6	0.305	0.000		1.4		1.3	0.010	0.355

Forty-seven patients in the T2DM cohort and 872 patients in the control cohort were excluded because they met the index event more than 20 years ago. Continuous variables are reported as mean ± SD, and categorical variables as n (%).

BMI indicates body mass index; n, number; N/A, not applicable; SD, standard deviation; T2DM, type 2 diabetes mellitus.

*P* < 0.05 indicates statistical significance.

**TABLE 3.** Comparative analysis for estradiol HRT vs no estradiol HRT

Demographics		Before propensity score matching						After propensity score matching					
		Estradiol HRT (*n* = 549,217)		No HRT (*n* = 1,261,045)				Estradiol HRT (*n* = 549,028)		No HRT (*n* = 549,028)			
		mean ± SD	% of cohort	Mean ± SD	% of cohort	Standard difference	*P* value	Mean ± SD	% of cohort	Mean ± SD	% of cohort	Standard difference	*P* value
AI	Age at Index	60.9 ± 9.5	100.0	59.0 ± 9.4	100.0	0.201	0.000	60.9 ± 9.4	100.0	60.9 ± 9.4	100	0.003	0.181
F	Female		100.0		100.0	N/A	N/A		100.0		100	N/A	N/A
2106-3	White		76.1		66.6	0.210	0.000		76.1		76.10	0.001	0.634
2186-5	Not Hispanic or Latino		71.9		65.7	0.133	0.000		71.8		71.97	0.003	0.152
2054-5	Black or African American		8.6		14.2	0.177	0.000		8.6		8.65	0.000	0.884
2135-2	Hispanic or Latino		5.9		8.0	0.083	0.000		5.9		5.78	0.005	0.017
2028-9	Asian		4.5		6.1	0.072	0.000		4.5		4.47	0.000	0.825
2131-1	Other race		3.0		3.0	0.001	0.570		3.0		2.96	0.000	0.960
1002-5	American Indian or Alaska Native		0.3		0.3	0.001	0.589		0.3		0.32	0.001	0.736
2076-8	Native Hawaiian or Other Pacific Islander		0.3		0.8	0.074	0.000		0.3		0.27	0.000	0.912
M	Male		0.0		0.0	N/A	N/A		0.0		0	N/A	N/A
Diagnoses													
E78	Disorders of lipoprotein metabolism and other lipidemias		39.8		32.5	0.153	0.000		39.8		39.77	0.000	0.800
I10	Essential (primary) hypertension		34.8		33.1	0.037	0.000		34.8		34.83	0.000	0.995
K70-K77	Diseases of liver		8.2		6.2	0.077	0.000		8.2		8.21	0.000	0.970
J44	Other chronic obstructive pulmonary disease		4.0		3.8	0.014	0.000		4.0		3.96	0.003	0.093
Z72.0	Tobacco use		2.3		2.4	0.003	0.047		2.3		2.27	0.003	0.164
F10	Alcohol related disorders		1.9		1.6	0.023	0.000		1.9		1.85	0.004	0.044
Medications													
40048	Carboplatin		0.2		0.3	0.004	0.018		0.2		0.21	0.008	0.000
2555	Cisplatin		0.1		0.1	0.013	0.000		0.1		0.11	0.008	0.000
Labs													
9084	BMI percentile		1.0		0.9	0.011	0.000		1.0		0.92	0.004	0.030

17,070 patients in the estradiol HRT cohort and 84,043 patients in the control cohort were excluded because they met the index event more than 20 years ago. Continuous variables are reported as mean ± SD, and categorical variables as n (%).

BMI indicates body mass index; HRT, hormone replacement therapy; n, number; N/A, not applicable; SD, standard deviation; T2DM, type 2 diabetes mellitus.

*P* < 0.05 indicates statistical significance.

All outcomes were assessed over a follow-up period extending ≤20 years from the index date. Risk ratio (RR) and odds ratio (OR) were calculated with 95% confidence intervals (CI) for each outcome. Statistical significance was defined using a *P* value of 0.05. Wald tests were used for hypothesis testing. Data analytics were performed within the TriNetX platform using its integrated analytics and validated tools.

### Ethical Considerations

This study lies under the jurisdiction of the University of Texas Medical Branch Institutional Review Board, which determined that studies utilizing the TriNetX platform have met the criteria for exemption from institutional review board review under US Federal Regulation 45 CFR 46.104(d) (5). The exemption determination was issued on April 16, 2020. In addition, all data accessed through the TriNetX platform was de-identified in accordance with the standards of TriNetX LLC and the Health Insurance Portability and Accountability Act.

## RESULTS

### Cohort Selection

After applying the criteria outlined for propensity score match in addition to excluding individuals with an otosclerosis diagnosis ≥20 years before index, 3 cohorts were formed to evaluate associations of each exposure with otosclerosis: obesity, T2DM, and estradiol HRT for menopause. Among data extracted from the TriNetX Research Network for analysis of obesity, we identified 99,455 patients with a diagnosis of obesity and 619,171 patients without. Among these patients, 82 individuals with obesity and 787 individuals without were excluded as they met the index event ≥20 years before data analysis (Table 1). In the T2DM cohorts, 16,569 patients with a diagnosis of T2DM and 106,098 patients with no recorded diagnosis of T2DM were identified. Forty-seven individuals with T2DM and 872 individuals without were excluded as they met the index event ≥20 years ago (Table 2). In the estradiol HRT cohorts, 549,217 patients with recorded estradiol HRT and 1,261,045 patients with no recorded estradiol HRT were identified. 17,070 individuals with recorded estradiol HRT and 84,043 individuals with no recorded HRT were excluded as they met the index event ≥20 years ago (Table 3).

### Characteristics Before and After Propensity Match: Exposure 1. Obesity

Before propensity matching, female sex comprised 63.4% of the cohort with obesity compared with 53.6% in controls (standardized mean difference [SMD] = 0.199, *P* < 0.0001). Male sex comprised 35.0% of individuals with obesity compared with 43.5% in control groups (SMD = 0.176, *P* < 0.0001). Demographics showed notable imbalances, with ethnicity characterized as not Hispanic or Latino, 71.5% in individuals with obesity, 63.9% in controls (SMD = 0.163, *P <* 0.0001). Black or African American race, obesity 21.7%, 11.8% controls (SMD = 0.267, *P <* 0.0001). Asian race, in obesity 2.1%, 4.4% in controls (SMD = 0.134, *P <* 0.0001). For diagnoses, disorders of lipoprotein metabolism and lipidemia were present in 44.7% of the obesity cohort and 19.8% in controls (SMD = 0.552, *P* < 0.0001), primary hypertension, 42.1% in obesity, 17.2% in controls (SMD = 0.566, *P* < 0.0001), diseases of liver, 11.7% in obesity, 3.5% in controls (SMD = 0.316, *P* < 0.0001), tobacco use, 5.5% obesity, 2.5% in controls (SMD = 0.153, *P* < 0.0001), other chronic obstructive pulmonary disease, 5.3% obesity, 2.2% controls (SMD = 0.163, *P* < 0.0001).

After propensity matching, 34,843 individuals with a diagnosis of obesity were matched to 34,843 controls. All baseline characteristics achieved a standardized difference ≤0.1. Mean age ± SD was 43.1 ± 20.2 for obesity versus 45.1 ± 20.8 for control (SMD = 0.093, *P* < 0.0001). Female sex: 61.6% obesity, 61.4% control (SMD = 0.004, *P* = 0.570); male: 36.5% obesity, 36.7% control (SMD = 0.003, *P* = 0.741) (Table 1).

### Characteristics Before and After Propensity Match: Exposure 2. T2DM and Otosclerosis

Before propensity matching, imbalanced covariates included age at index (mean ± SD) in individuals with T2DM was 59.2 ± 16.3, and in controls was 41.6 ± 19.0 (SMD = 0.991, *P* < 0.0001). Ethnicity reported as not Hispanic or Latino: 72.4% T2DM, 65.4% in controls (SMD = 0.151, *P* < 0.0001), Hispanic or Latino: 12.4% T2DM, 9.3% controls (SMD = 0.101, *P* < 0.0001). Race reported as Black or African American: 19.5% in T2DM, 13.4% in controls (SMD = 0.167, *P* < 0.0001). For diagnoses, primary hypertension: 69.9% T2DM, 21.2% controls (SMD = 1.217, *P* < 0.0001), disorders of lipoprotein metabolism and lipidemia: 68.9% T2DM, 21.2% controls (SMD = 1.095, *P*< 0.0001), diseases of liver: 17.6% T2DM, 4.2% controls (SMD = 0.440, *P* < 0.0001), other chronic obstructive pulmonary disease: 10.7% T2DM, 2.1% control (SMD = 0.357, *P* < 0.0001), tobacco use: 7.5% T2DM, 2.8% control (SMD = 0.214, *P* < 0.0001), alcohol related disorders: 4.4% T2DM, 2.4% controls (SMD = 0.111, *P* < 0.0001).

After propensity matching, 15,702 individuals with a diagnosis of T2DM were matched to 15,702 controls. All baseline characteristics achieved a standardized difference ≤ 0.1 except for diseases of liver (17.6% T2DM, 10.4% control, SMD = 0.208, *P* < 0.0001) and other chronic pulmonary disease (10.3% T2DM, 6.8% control, SMD = 0.127, *P* < 0.0001). Mean age ± SD was 58.6 ± 16.4 for T2DM versus 59.0 ± 16.5 for control (SMD = 0.024, *P* < 0.05). Female sex: 55.9% T2DM, 56.1% control (SD = 0.004, *P* = 0.708); male: 42.2% T2DM, 41.9% control (SMD = 0.006, *P* = 0.575) (Table 2).

### Characteristics Before and After Propensity Match: Exposure 3. Estradiol HRT and Otosclerosis

Before propensity matching, imbalanced covariates included age at index (mean ± SD), which was 60.9 ± 9.5 in individuals with estradiol HRT and 59.0 ± 9.4 in those without estradiol HRT (SMD = 0.201, *P* < 0.0001). The cohort was 100% female. Reported race as white was 76.1% in HRT, 66.6% no HRT (SMD = 0.210, *P* < 0.0001), black or African American: 8.6% HRT, 14.2% no HRT (SMD = 0.177, *P* < 0.0001). Reported ethnicity as not Hispanic or Latino was 71.9% HRT, 65.7% no HRT (SMD = 0.133, *P* < 0.0001). For diagnoses, disorders of lipoprotein metabolism and other lipidemia, 39.8% in HRT, 32.5% in no HRT (SMD = 0.153, *P* < 0.0001).

After propensity matching, 549,028 individuals with estradiol therapy for menopause were matched to 549,028 controls. All baseline characteristics achieved a standardized difference ≤0.1. Mean age ± SD was 60.9 ± 9.4 T2DM versus 60.9 ± 9.4 control (SMD = 0.003, *P* = 0.181) (Table 3).

### Outcomes After Propensity Score Matching

**FIG 1.**
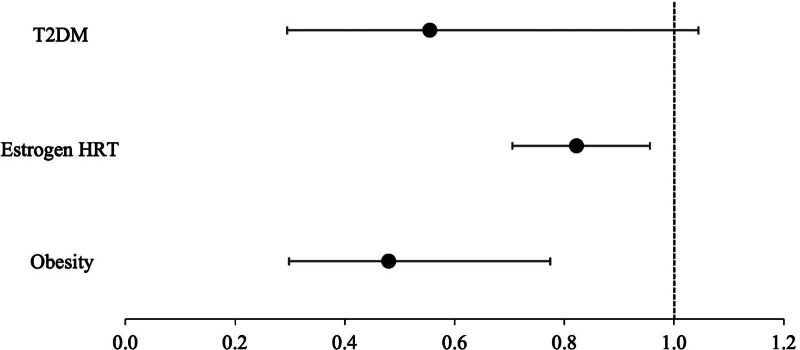
Forest plot of relative risk for otosclerosis across matched cohorts. Graph representative of relative risk and 95% confidence intervals for risk of otosclerosis development with obesity, T2DM, and estradiol HRT. Vertical dashed line at *x* = 1 indicates no association. HRT indicates hormone replacement therapy; T2DM, type 2 diabetes mellitus.

Exposure 1. Obesity: after selection and propensity score match, 34,843 individuals with a diagnosis of obesity and 34,843 controls were indexed for otosclerosis. Ninety-eight patients with a diagnosis of obesity and 136 patients with no diagnosis of obesity were excluded from the results because they had the outcome before the time window. Among patients with obesity, 25 individuals developed otosclerosis during follow-up with a cumulative incidence of 0.025%; in controls, 52 individuals developed otosclerosis with a cumulative incidence of 0.053%. Absolute risk difference was −0.078% (95% CI, −0.127% to −0.028%, *P* < 0.005). The RR for otosclerosis was 0.48 (95% CI, 0.298–0.774), indicating that patients with obesity had a 52% lower risk of developing otosclerosis compared with matched controls (Fig. 1). The OR was 0.480 (95% CI, 0.297–0.773) (Table 4).

**TABLE 4.** Measures of association

	Cohort statistics			Risk difference				Risk ratio		Odds ratio	
Cohort	Patients in cohort	Patients with outcome	Risk	Risk difference	95% CI	z	*P* value	Risk ratio	95% CI	Odds ratio	95% CI
Obesity	34745	25	0.072%	−0.078%	(−0.127% to −0.028%)	−3.083	0.002	0.480	(0.298–0.774)	0.480	(0.297–0.773)
No Obesity	34707	52	0.150%								
T2DM	15641	15	0.096%	−0.077%	(−0.157% to 0.004%)	−1.854	0.064	0.555	(0.295–1.044)	0.555	(0.295–1.044)
No T2DM	15638	27	0.173%								
Estradiol HRT	548161	303	0.055%	−0.012%	(−0.021% to −0.003%)	−2.539	0.011	0.822	(0.705–0.956)	0.822	(0.705–0.956)
No Estradiol HRT	548472	369	0.067%								

Ninety-eight patients in the obesity Cohort and 136 patients in the control obesity cohort were excluded from the results because they had the outcome before the time window. Sixty-one patients in the T2DM cohort and 64 patients in the T2DM control cohort were excluded from the results because they had the outcome before the time window. Eight hundred sixty-seven patients in the estradiol HRT cohort and 556 patients in the estradiol HRT control cohort were excluded from the results because they had the outcome before the time window.

HRT indicates hormone replacement therapy; T2DM, type 2 diabetes mellitus.

*P* < 0.05 indicates statistical significance.

Exposure 2. T2DM and otosclerosis: after selection and propensity score match, 15,702 individuals with a diagnosis of T2DM and 15,702 controls were indexed for otosclerosis. Sixty-one patients in the T2DM cohort and 64 patients in the no T2DM cohort were excluded from the results because they had the outcome before the time window. Among patients with T2DM, individuals developed otosclerosis during follow-up with a cumulative incidence of 0.1%; in controls, 27 individuals developed otosclerosis with a cumulative incidence of 0.17%. Absolute risk difference was −0.077% (95% CI, −0.157% to 0.004%, *P* = 0.064). The RR for otosclerosis was 0.555 (95% CI, 0.295–1.044), suggesting a 44% lower risk of otosclerosis in patients with T2DM compared with matched controls (Fig. 1). The OR was 0.555 (95% CI, 0.295–1.044) (Table 4).

Exposure 3. Estradiol HRT and otosclerosis: after selection and propensity score match, 15,702 individuals with estradiol HRT and 15,702 controls were indexed for otosclerosis. Totally, 867 patients in the estradiol HRT cohort and 556 patients in the no estradiol HRT cohort were excluded from the results because they had the outcome before the time window. Among patients with estradiol HRT, 303 individuals developed otosclerosis during follow-up with a cumulative incidence of 0.06%; in controls, 369 individuals developed otosclerosis with a cumulative incidence of 0.07%. Absolute risk difference was −0.012% (95% CI, −0.021% to −0.003%, *P* < 0.05). The RR for otosclerosis was 0.822 (95% CI, 0.705–0.956), suggesting that estradiol users experienced an 18% lower risk of incidence of otosclerosis compared with matched controls (Fig. 1). The OR was 0.822 (95% CI, 0.705–0.956]) (Table 4).

## DISCUSSION

### Summary of Key Findings

This multicenter retrospective cohort study sought to elucidate the association between elevated-estrogenic states, including obesity, T2DM, and postmenopausal HRT, and the development of otosclerosis. After detailed 1:1 propensity score matching and exclusion of patients with preexisting otosclerosis, obesity, and estradiol HRT were each independently associated with a significant reduction in the risk for development of otosclerosis, with a greater effect size seen in obesity and otosclerosis. Conversely, the inverse association between T2DM and otosclerosis did not reach the significance threshold. Together, our findings lend epidemiologic support to the hypothesis that systemic estrogen exposure seen in estrogenic states may serve to mitigate the abnormal bone remodeling and metabolism seen in otosclerosis.

### Comparison with Prior Literature

Prior epidemiological studies examining hormonal influences on otosclerosis have produced conflicting results. Early reports suggest a detrimental effect of combined estrogen-progestin oral contraceptives on hearing, whereas later studies demonstrated an association between higher serum estradiol levels and improved auditory thresholds. It is important to note that the otic capsule demonstrates unique bone metabolism distinct from other bones in the human body, characterized by an isolated lacuna-canalicular network with a tightly regulated osteoprotegerin/receptor activator of nuclear factor kappa-B ligand ratio. This balance modulates osteoclast activity locally, and estrogen’s effects on this microenvironment may differ from other bone remodeling pathways (2).

Our observation that estradiol HRT is associated with a reduced risk of otosclerosis aligns with prior literature that characterizes a beneficial role of estrogen on one’s overall hearing health (5). Therefore, our findings bolster a putative protective role of estrogen against the development of otosclerosis. While we did not find a significant relationship between T2DM and risk for otosclerosis, this is not unexpected considering previous literature, which outlines the complex relationship between estrogen signaling and the development of T2DM (9). Previously, a relationship has been demonstrated between T2DM and changes in middle ear ossicular joints, including increased hyalinization and altered joint morphology (10); however, the mechanistic link between the middle ear and T2DM has yet to be fully elucidated. Finally, the strong protective association against otosclerosis in individuals with obesity echoes prior literature, which demonstrates higher bone mineral density in individuals with obesity, both due to increased mechanical force and an increase in androgens from adipose tissue (11).

Collectively, these findings support a protective role of circulating estrogen in maintaining the delicate homeostasis of bone metabolism within the otic capsule and may help explain the increased frequency of otosclerosis during periods of significant hormonal fluctuation, such as pregnancy and menopause.

### Strengths and Limitations

Key strengths of this study include the large, diverse sample size, use of electronic medical records, and our rigorous use of 1:1 propensity matching to adjust for potential confounding variables. The multicenter design leveraging the TriNetX Research Platform allows for broad geographic and demographic representation, enhancing the generalizability of our findings.

As with all studies utilizing administrative or billing databases, diagnostic coding accuracy is an inherent limitation. ICD-10 coding of otosclerosis (H80) may variably capture both histologically confirmed otospongiosis and clinically diagnosed stapes fixation, as surgical confirmation is not uniformly coded. Previous work has demonstrated significant potential for error when relying on administrative data for diagnostic purposes (12). Additionally, otosclerosis is frequently a clinical diagnosis supported by audiometric findings and, when applicable, is confirmed intraoperatively during stapedectomy. Diagnostic consistency across institutions may vary, underscoring a need for multi-modal validation studies incorporating audiometric or histopathologic data. Our use of a large, multicenter dataset and propensity matching across multiple institutions likely mitigates some variability in coding practices.

Additional limitations include adherence to HRT, chronic glycemic control, vitamin D levels, and genetic predisposition to otosclerosis may influence risk assessment in this study. Obesity, T2DM glycemic control, and adherence to HRT can fluctuate, and our findings are not able to account for these confoundings. Last, it is important to note that clinical classification of obesity is based on BMI and does not accordingly account for individuals with increased muscle mass, or differentiate between central, visceral, and subcutaneous adiposity (13).

Taken together, these strengths and limitations highlight the robustness of our analytical approach and the need for future prospective studies incorporating audiometric data, serum hormone panels, and genotypic information to validate these findings.

### Clinical Implications of Findings and Future Directions

Although the global incidence of otosclerosis is relatively low, the relative risk reductions observed here suggest a potential for clinicians to consider hormonal and metabolic modifications when counseling patients at a higher baseline risk for the development of otosclerosis, as the condition has a strong genetic component within families (14). Particular attention should be given to lean, postmenopausal women with frequent hearing screenings in order to prevent estrogen-associated hearing loss (15). Our findings reinforce the plausibility of estrogen-mediated protection within the otic capsule, consistent with prior evidence linking estrogen to bone remodeling and anti-inflammatory signaling in this region. The modest protective role of estradiol HRT outlined in this study further supports a nuanced relationship between systemic hormone therapy and otosclerotic bone metabolism, suggesting a potential therapeutic approach for certain susceptible individuals, particularly as they transition through periods of increased hormonal vulnerability, such as menopause. Similarly, the inverse trends observed in obesity and T2DM align with prior models proposing that higher circulating estrogen levels and adipose-derived hormone activity may exert protective influences on bone turnover.

As estradiol therapy presents with significant thrombotic and oncogenic risks (16), further investigation could outline a potential local delivery of estrogen into the otic capsule for those at risk. In addition, future studies may serve to incorporate longitudinal analysis of audiometry, serum hormone panels including estrogen, progesterone, and prolactin, and genotyping to clarify the biomechanical interaction of estrogen and otosclerosis. Collectively, these observations support the interpretation that estrogenic and metabolic factors exert meaningful, biologically significant influences on otic capsule remodeling, encouraging future translational studies that bridge epidemiologic and molecular data.

## CONCLUSIONS

Our retrospective cohort study demonstrated that obesity and estradiol HRT were independently associated with a reduced risk for the development of otosclerosis, whereas a nonsignificant but slightly protective trend was identified in patients with T2DM. These findings, together with prior literature, underscore a biologic model in which estrogen and metabolic states modulate otic capsule homeostasis. These results suggest a protective role of estrogen against the development of otosclerosis and prompt consideration of these relationships for clinical treatment and therapeutic intervention for individuals with and at risk for otosclerosis. While the observational nature of this study prevents determining causation, these findings have generated intriguing testable hypotheses for biomechanical and interventional research aimed at preventing and treating otosclerosis hearing loss.

## FUNDING SOURCES

This research was supported by the UTMB Institute for Translational Sciences, supported in part by a Clinical and Translational Science Award (UL1 TR001439) from the National Center for Advancing Translational Sciences at the National Institutes of Health (NIH). The content is solely the responsibility of the authors and does not necessarily represent the official views of the NIH.

## CONFLICT OF INTEREST

None declared.

## DATA AVAILABILITY

The data that support the findings of this study are openly available in TriNetX at live.trinetx.com.

## References

[1] World Health Organization. International Statistical Classification of Diseases and Related Health Problems, 10th Revision (ICD-10). Geneva, Switzerland: World Health Organization; 2016. H80-Otosclerosis

[2] Horner KC. The effect of sex hormones on bone metabolism of the otic capsule – an overview. Hear Res. 2009;252:56–60. 19121641 10.1016/j.heares.2008.12.004

[3] Kim S. The association between serum estradiol level and hearing sensitivity in postmenopausal women. Obstet Gynecol. 2002;99:726–730. 11978279 10.1016/s0029-7844(02)01963-4

[4] Handgraaf S Dusaulcy R Visentin F Philippe J Gosmain Y. 17-β estradiol regulates proglucagon-derived peptide secretion in mouse and human α- and L Cells. JCI Insight. 2018;3:e98569. 29618657 10.1172/jci.insight.98569 PMC5928861

[5] Kilicdag EB Yavuz H Bagis T Tarim E Erkan AN Kazanci F. Effects of estrogen therapy on hearing in postmenopausal women. Am J Obstet Gynecol. 2004;190:77–82. 14749639 10.1016/j.ajog.2003.06.001

[6] Erdem D Şevik Eliçora S Güven B. GPER-1 and sex-hormone levels in patients with otosclerosis. Am J Otolaryngol. 2020;41:102442. 32144019 10.1016/j.amjoto.2020.102442

[7] World Health Organization. International Statistical Classification of Diseases and Related Health Problems, 10th Revision (ICD-10). Geneva, Switzerland: World Health Organization; 2016. E66-Obesity

[8] World Health Organization. International Statistical Classification of Diseases and Related Health Problems, 10th Revision (ICD-10). Geneva, Switzerland: World Health Organization; 2016. E11-Type 2 diabetes mellitus

[9] Yan H Yang W Zhou F. Estrogen improves insulin sensitivity and suppresses gluconeogenesis via the transcription factor Foxo1. Diabetes. 2019;68:291–304. 30487265 10.2337/db18-0638 PMC6341301

[10] Shimura T Keskin Yilmaz N Rajan D Cureoglu S Da Costa Monsanto R. Middle ear ossicular joint changes in type 2 diabetes mellitus: a histopathological study. Laryngoscope. 2024;134:2871–2878. 38174760 10.1002/lary.31257 PMC11078616

[11] Rinonapoli G Pace V Ruggiero C. Obesity and bone: a complex relationship. Int J Mol Sci . 2021;22:13662. 34948466 10.3390/ijms222413662 PMC8706946

[12] Zelicha H Bell DS Chen Y Livingston EH. Potential for error when relying on administrative data. Br J Surg. 2025;112. doi:10.1093/bjs/znaf139. 10.1093/bjs/znaf139 PMC12268322 40673449

[13] Muscogiuri G Verde L Vetrani C Barrea L Savastano S Colao A. Obesity: a gender-view. J Endocrinol Invest. 2024;47:299–306. 37740888 10.1007/s40618-023-02196-z PMC10859324

[14] Thys M Van Camp G. Genetics of otosclerosis. Otol Neurotol. 2009;30:1021–1032. 19546831 10.1097/MAO.0b013e3181a86509

[15] Curhan SG Eliassen AH Eavey RD Wang M Lin BM Curhan GC. Menopause and postmenopausal hormone therapy and risk of hearing loss. Menopause. 2017;24:1049–1056. 28486246 10.1097/GME.0000000000000878 PMC5570623

[16] Baik SH Baye F McDonald CJ. Use of menopausal hormone therapy beyond age 65 years and its effects on women’s health outcomes by types, routes, and doses. Menopause. 2024;31:363–371. 38595196 10.1097/GME.0000000000002335 PMC11465799

